# Gold Nanoparticle-Induced Cell Death and Potential Applications in Nanomedicine

**DOI:** 10.3390/ijms19030754

**Published:** 2018-03-07

**Authors:** Hainan Sun, Jianbo Jia, Cuijuan Jiang, Shumei Zhai

**Affiliations:** 1 School of Chemistry and Chemical Engineering, Shandong University, Jinan 250100, China; sunhainan1986@126.com (H.S.); jiajianbo03@gmail.com (J.J.); 2 School of Environmental Science and Engineering, Guangzhou University, Guangzhou 510006, China; 3 School of Environmental Science and Engineering, Shandong University, Jinan 250100, China; cjjiang@sdu.edu.cn

**Keywords:** gold nanoparticles, cell death, proliferation, apoptosis, necrosis, autophagy

## Abstract

Cell death is crucial to human health and is related to various serious diseases. Therefore, generation of new cell death regulators is urgently needed for disease treatment. Nanoparticles (NPs) are now routinely used in a variety of fields, including consumer products and medicine. Exhibiting stability and ease of decoration, gold nanoparticles (GNPs) could be used in diagnosis and disease treatment. Upon entering the human body, GNPs contact human cells in the blood, targeting organs and the immune system. This property results in the disturbance of cell function and even cell death. Therefore, GNPs may act as powerful cell death regulators. However, at present, we are far from establishing a structure–activity relationship between the physicochemical properties of GNPs and cell death, and predicting GNP-induced cell death. In this review, GNPs’ size, shape, and surface properties are observed to play key roles in regulating various cell death modalities and related signaling pathways. These results could guide the design of GNPs for nanomedicine.

## 1. Introduction

Apoptosis, autophagy, necroptosis, aponecrosis, pyroptosis, and necrosis are major cell death modalities [1,2,3,4,5]. The modalities differ with regards to trigger, timing, degree of regulation, and key regulators. Apoptosis is used by multicellular organisms to remove unwanted cells through dedicated, controlled extrinsic and intrinsic pathways [6,7]. Apoptosis occurs during body development, aging, and as a homeostatic mechanism to hold cell populations. Autophagy is a self-digesting process to eliminate long-lived proteins and damaged organelles [8]. Necroptosis, which can also be called “programmed necrosis”, is initiated by activating the death receptor with stimuli, among which receptor-interacting protein kinases 1 and 3 are frequently involved [3]. Pyroptosis is a caspase 1-dependent form of cell death. Caspase 1 activation leads to cytokine processing and pore formation in cell membranes, which leads to cellular ionic gradient dissipation, osmotic pressure enhancement, water influx, cell swelling and, eventually, osmotic lysis and release of cytokines [5]. 

Cell death plays an important role in maintaining human health, and is associated with various diseases, including neurodegenerative diseases, ischemic damage, autoimmune disorders, cancer [9,10,11,12,13,14], aging [15], type 2 diabetes [16], and atherosclerosis [17,18]. For example, inhibition of apoptosis is associated with cancer, autoimmune disorders, and viral infections. Therefore, increasing apoptosis will inhibit carcinogenesis and be helpful in treating autoimmune disorders and viral infections. However, excessive apoptosis is associated with AIDS, neurodegenerative diseases, myelodysplastic syndromes, ischemia-associated injury, and toxin-induced liver disease. Therefore, reducing apoptosis will be helpful to cure these diseases [10]. Necrosis is accompanied with inflammation effect, therefore, people try not to elicit necrosis in disease treatment, including cancer therapy. Autophagy is a cytoprotective mechanism to prevent and treat various diseases. Appropriately increasing autophagy could delay aging, suppress tumor, and be helpful in treating type 2 diabetes, atherosclerosis, and neurodegenerative diseases [16,17,18]. Considering the important role of cell death in human diseases, it could be a novel therapeutic target for the treatment of these diseases. Developing cell death regulators provides a new strategy for disease therapy.

Nanomaterials have a similar scale to biological macromolecules, which may cause them to directly affect biological systems. Therefore, nanomaterials could be used in biomedical sciences [19,20,21,22,23,24]. Due to the stability, size-tunable surface plasmon resonance, fluorescence, and easy-surface functionalization, gold nanoparticles (GNPs) could be applied to imaging diagnosis [25], drug delivery [26], radiosensitization [27,28], and photothermal therapy [29,30]. For drug delivery, GNPs were used as a compatible vector to deliver drugs and target ligands. For radiosensitization and photothermal therapy purposes, biocompatible GNPs could kill cancer cells under external stimuli. Therefore, in most cases, GNPs seem only to act as a “cargo” and “assist” in biomedical areas. However, because GNPs could also act as cell death regulators, they may synergistically treat diseases along with loaded drugs and external stimuli.

GNPs’ physicochemical properties, including size, shape, and surface properties (charge, ligand density, and hydrophobicity), were considered to affect biological activity in the past, such as protein absorption and cellular uptake [31,32,33,34]. Considering GNPs’ potential application in cell death regulation, precisely understanding the relationship between GNP properties and cell death will assist in designing and optimizing GNPs for biomedical applications.

In this review, we summarized how GNP properties affect cell death, and the potential use in disease treatment, primarily focusing on apoptosis, necrosis, autophagy, and associated molecular mechanisms.

## 2. Determination of Cell Proliferation by GNPs

GNP size tailors their direct effect on biological systems by dictating cell proliferation. Physically produced GNPs, ranging from 2 to 40 nm in size, inhibited proliferation of J774 A1 macrophages significantly, with increasing inhibition being observed as GNP size decreased [35]. Another paper reported that GNPs with a diameter of 10 nm did not affect proliferation of MG63 osteoblast-like cells at concentrations of 1 ppm and 10 ppm [36]. The proliferation of human periodontal ligament cells (hPDLCs) and human periodontal ligament stem cells (hPDLSCs) was investigated with GNPs of various diameters (20, 40, 60, and 80 nm). Only 60 nm-sized GNPs could effectively promote hPDLC and hPDLSC proliferation, whereas the other three GNPs (20, 40, and 80 nm) inhibited proliferation [37]. The results help to elucidate the advantage of GNPs in proliferation dictation of macrophages and stem cells, demonstrating the potential application of differently sized GNPs in the treatment of immunological and dental diseases. 

In addition to spherical GNPs, gold nanorods (GNRs) have been thoroughly studied for a variety of potential medical applications, including cancer diagnostics and therapeutics [30]. GNRs coated with different ligands induced unique responses in human keratinocyte (HaCaT) cells [38]. Thiolated polyethylene glycol (PEG)-GNRs and mercaptohexadecanoic acid (MHDA)-GNRs exhibited minimal effects on cell proliferation, whereas cetyltrimethylammonium bromide (CTAB)-GNRs reduced cell proliferation significantly, due to inherent toxicity of the cationic ligands to cells. In another paper, 30 and 50 nm PEG-GNRs reduced cell viability and caused DNA damage in human lymphocytes, with smaller GNRs inducing more cyto- and genotoxic effects than did larger GNRs [39]. 

Sodium citrate, which is a commonly used stabilizer during GNPs synthesis, could modulate proliferation. For example, different sized citrate-GNPs affected proliferation. Citrate-GNPs (5 nm) inhibited proliferation of three multiple myeloma (MM) cell lines (OPM-1, RPMI-8266, and U-266) through cell cycle arrest in the G1 phase via upregulation of cell cycle proteins p21 and p27 [40]. Citrate-GNPs (14 nm) are responsible for abnormal actin filaments and extracellular matrix constructs in human dermal fibroblasts, which reduces cellular proliferation [41]. In addition, the presence of citrate-GNPs increased population doubling times of human adipose-derived stromal cells (ADSCs) in a size- and concentration-dependent manner [42]. Furthermore, it was observed that the presence of excess citrate on the surface of GNPs, rather than the particle size, reduced viability and proliferation of epithelial cells, including human alveolar cell lines A549 and NCIH441 [43]. The same effects were detected after exposure of endothelial cells to the same nanoparticles, although endothelial cells were less sensitive to GNPs than epithelial cells. This finding suggested the amount of sodium citrate should be tuned to a level where particle stability and their biomedical applications in MM and lung cancer treatment are guaranteed [44].

In addition to cell proliferation dictated by GNPs alone, GNPs could also block growth factor-mediated cell proliferation [45,46]. GNPs (50 nm) blocked the VEGF- and IL-1β-induced proliferation in bovine retinal pigment epithelial cells (BRPEs) by suppressing the Src kinase pathway, showing potential applications in treatment of ocular diseases, such as proliferative vitreoretinopathy [45]. GNPs (5 nm) inhibited VEGF165-induced HUVEC proliferation through binding to the heparin-binding domain of VEGF165, which inhibited VEGF165-induced signaling. This finding demonstrated that GNPs are a feasible choice for determining growth factor-mediated proliferation [46].

Cytotoxicity and GNP-induced inhibition of cell proliferation could be ascribed to apoptosis, necrosis, and autophagy mechanisms, which are demonstrated in detail in the following sections.

## 3. GNP-Induced Apoptosis

There are two types of pathways mediating apoptosis: extrinsic and intrinsic pathways. Extrinsic pathways are mediated by death receptor superfamily proteins, such as CD95 and tumor necrosis factor receptor I. These death receptors will be activated when death signals are received, resulting in cell apoptosis. Intrinsic pathways are mediated by mitochondria or the endoplasmic reticulum (ER) [47,48]. Apoptosis could be determined at the genetic, protein, and cytological levels. At the genetic level, terminal transferase-mediated deoxyuridine triphosphate (dUTP) nick end labeling (TUNEL) is commonly employed to detect apoptosis-induced DNA fragments [49]. Apoptosis-related genes include pro-apoptotic Bax, caspase-3, caspase-9, TNFSF10, ANXA5, CASP1, CASP8, EGR1, c-jun, p53, nuclear receptor subfamily 4 group A member 1 (NR4A1), phorbol-12-myristate-13-acetate-induced protein 1 (PMAIP1), and reticulon 4 (RTN4), and anti-apoptotic Bcl-2, 24-dehydrocholesterol reductase (DHCR24), and RB1-inducible coiled-coil 1 (RB1CC1) [50,51]. The upregulation of pro-apoptotic genes and downregulation of anti-apoptotic genes indicates that apoptosis may occur. At the protein level, apoptosis-related proteins include pro-apoptotic caspase 3/7/8/9, cytochrome c, Bax, and p53, and anti-apoptotic Bcl-2 [47]. Western blotting and immunofluorescence could detect caspase upregulation, Bax translocation into mitochondria, cytochrome c release into cytoplasm, and Bcl-2 downregulation, which all indicate apoptosis. At the cytological level, apoptosis could be conveniently determined by AO/EB or annexin V/PI/7AAD-dual staining. Electron and light microscopy examination of transmission electron microscopy (TEM) images and histological tissue samples could also yield visual apoptosis-related information [9].

Apoptosis levels are related to GNP size. Citrate-decorated GNPs (13 nm) induced apoptosis of rabbit articular chondrocytes, while 3 and 45 nm-sized citrate-GNPs did not affect cell viability [52]. GNPs (15 nm) induced more apoptosis than 5 nm-sized GNPs at high concentrations (10 and 100 μg/mL) in human peripheral blood lymphocytes (PBL) and murine macrophages (Raw264.7) [53]. For PEG-decorated GNPs, 4 and 100 nm-sized GNPs induced similar levels of apoptosis in liver tissue after intravenous administration to BALB/c mice (4.26 mg/kg, body weight) [51]. Therefore, GNPs measuring approximately 15 nm in diameter seemed to induce the highest apoptosis level, showing potential use in joint and immunological disease treatment.

**Figure 1:**
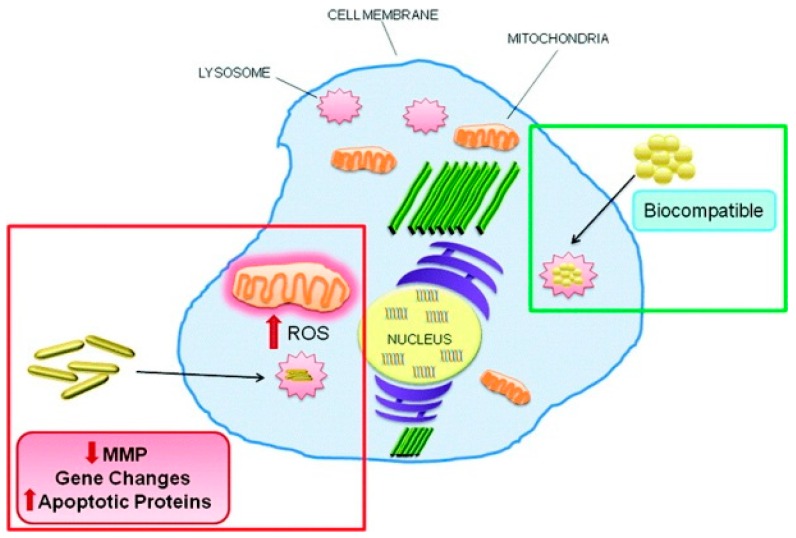
Long polyethylene glycol-capped gold nanorods induced significant reactive oxygen species (ROS) production, disrupted the mitochondrial membrane potential and elicited apoptosis, while mercaptopropane sulfonate-capped gold nanospheres did not show any of cytotoxic effects. Reprinted with permission from reference [50]. Copyright (2012) American Chemical Society.

GNP shape also affects apoptosis level. Compared to triangular- and spherical-shaped GNPs, hexagonal GNPs containing HS–C_2_H_4_–CONH–PEG–O–C_3_H_6_–COOH induced higher levels of reactive oxygen species (ROS) and pro-apoptotic markers, including Fas, caspase 3, and caspase 9, in Calu-3 epithelial cells [54]. Apoptosis induced by PEG-decorated gold nanorods (PEG-GNR, 16.7 nm × 43.8 nm) and mercaptopropane sulfonate (MPS)-decorated gold nanospheres (GNS-MPS, 20 nm) were evaluated in HaCaT cells. Compared to GNS-MPS, GNR-PEG caused significant ROS production and upregulation of apoptosis-related genes (TNFSF10, ANXA5, CASP1, and EGR1) and proteins (caspase 1) (Figure 1) [50]. Therefore, it seems that compared to spherical-shaped GNPs, hexagonal GNPs and GNRs are more liable to induce apoptosis.

GNP surface chemistry is another factor that could determine apoptosis. Trimethylammoniumethanethiol (TMAT), mercaptoethanesulfonate (MES), and mercaptoethoxyethoxyethanol (MEEE) decoration of 1.5 nm-sized GNPs provide positive, negative, and neutral charge, respectively. Positively charged TMAT-GNPs and negatively charged MES-GNPs increased caspase 3 expression and caused apoptosis in HaCaT cells, while neutral-charged MEEE-GNPs induced necrosis [55]. Another paper showed that negatively charged sodium polyacrylate-decorated GNPs (1–10 nm) induced apoptosis in freshly isolated human neutrophils, while positively charged poly(quaternary ammonium)-decorated GNPs did not elicit apoptosis. The different conclusions from those two papers may be due to different cell types, size, and ligand structure [56]. Ligand hydrophobicity of GNPs (20–25 nm) could determine apoptosis levels in A549 lung cancer cells. GNPs with the most hydrophobic ligand, dicationic cysteamine-conjugated lithocholic acid (DCaLC), elicited the highest level of apoptosis compared to GNPs with two other ligands, dicationic cysteamine-conjugated cholic acid (DCaC) and dicationic cysteamine-conjugated deoxycholic acid (DCaDC) [57]. The chirality of surface ligands also affected apoptosis. Compared to l-glutathione-decorated GNPs, d-glutathione-decorated GNPs induced a higher level of apoptosis, ROS generation, and mitochondrial membrane depolarization in MGC-803 cells [58]. Polyvinylpyrrolidone (PVP)-coated GNPs (7.4 nm ± 2.8 nm) activated caspase 3 and enhanced the ratio of apoptotic cells, as seen by annexin V/PI staining in HT-29 cells [59]. Citrate-coated GNPs (8, 15, and 40 nm) were observed to elicit apoptosis in HT29, SPEV, HL7702, and A549 cells [60,61,62]. In a comparative study, citrate-coated GNPs (40 nm) enhanced caspase 3 and Bax gene expression in a dose-dependent manner (4–1600 μg/L) in the head kidney of *Sparus aurata*, while PVP-coated GNPs (40 nm) showed a U-shaped response, with the highest level of caspase 3 and Bax gene expression induced at 80 μg/L [63]. Except for PVP and citrate decoration, NaBH_4_-, BSA-, imidazole-, and PEG-decorated GNPs could also elicit apoptosis. GNPs (29 nm) with NaBH_4_ as a reducing agent induced apoptosis in A549 cells, indicating that GNPs could act as a potential drug for lung cancer [64]. BSA-coated GNPs (1 nm) induced apoptosis in HepG-2 cells [65]. Also, 2-mercapto-1-methylimidazole-stabilized GNP (2.7 ± 0.7 nm)-induced neuronal apoptosis, as demonstrated by TUNEL assay and caspase-3 immunoreactivity [66]. PEG-coated GNPs (13 nm) accumulated in the liver and elicited apoptosis 7 days after injection, as seen by pathological examination and TUNEL assay analysis [67]. Therefore, GNPs with delicate decoration could be used in liver, lung, gastric, and colon cancer treatments.

GNPs decorated with metallic and non-metallic materials could also elicit apoptosis. Platinum-coated gold nanorods (25 nm × 75 nm) upregulated mRNA expression of apoptotic genes (bax, caspase-3, and caspase-9), downregulated anti-apoptotic gene bcl-2, and activated caspase-3 and caspase-9 enzymes in human breast carcinoma (MCF-7) cells [68]. The combination of mesoporous silica NPs on GNRs dictated apoptosis levels in MCF-7 cells, with Janus Au@mSiO_2_ NPs inducing the lowest level of apoptosis compared to bare gold nanorods and core–shell Au@mSiO_2_ NPs. Therefore, bare gold nanorods and core–shell Au@mSiO_2_ NPs may be more suitable than Janus Au@mSiO_2_ NPs for applications in biomedicine, including breast carcinoma treatment [69]. Gold–silver nanocages (107.4 nm) upregulated apoptosis-related genes in *Escherichia coli* cells [70]. 

GNP-induced apoptosis varied in different cell lines. GNRs (10 nm × 39 nm, 10 nm × 41 nm) elicited apoptosis in AGS cells (human gastric adenocarcinoma cells), but not in A549 cells [71]. GNPs (10–40 nm) induced apoptosis in Vero cells, but not in MRC-5 or NIH3T3 cells [72]. Also, it was observed that GNRs (50–60 nm × 20–30 nm) induced apoptosis in cancer cell lines MCF-7 and N87 by affecting lysosomes and mitochondria, while it showed a negligible impact on normal Chinese hamster ovary (CHO) and 293T cell lines, indicating GNR’s potential use in cancer treatment [73].

GNPs mainly elicited apoptosis through intrinsic pathways, including mitochondria- and ER-related pathways. Mitochondria-related apoptosis could be elicited by upstream ROS production. For example, ROS produced by platinum-coated gold nanorods (25 nm × 75 nm) and mesoporous silica nanoparticles on gold nanorods induced mitochondria-related apoptosis in human breast carcinoma (MCF-7) cells [68,69]. BSA-coated GNPs (1 nm) induced ROS-dependent apoptosis in HepG-2 cells [65]. Pretreatment with *N*-acetyl-l-cysteine (NAC) alleviated the apoptosis level induced by these GNPs, except for ROS scavengers, Bax translocation, cytochrome c release, upregulation of p53, downregulation of Bcl-2, and cleavage of poly(ADP-ribose) polymerase (PARP) could also confirm the mitochondria-related apoptosis mechanism. Citrate-coated GNPs (8 nm) elicited Bax translocation and cytochrome c release in human liver (HL7702) cells [61]. Citrate-coated GNPs (40 nm) elicited upregulation of p53, downregulation of Bcl-2, and cleavage of PARP in A549 cells [62]. In addition to the mitochondria pathway, GNPs (20 and 70 nm) could also activate ER-mediated apoptosis, as evidenced by activation of the three ER sensors IRE1, PERK, and ATF-6 in human neutrophils [74].

Based on the reported papers, apoptosis levels are affected by GNP properties, such as size, shape, and surface chemistry, as well as cell type (Table 1), demonstrating their potential applications in cancer, joint, and immunological disease treatments. As for the apoptosis mechanism, GNPs primarily induced apoptosis through the mitochondria pathway. The extrinsic apoptosis mechanism was not reported. 

## 4. GNP-Induced Necrosis

Necrosis could be elicited by activation of membrane receptors, such as death receptor (TNFR1) and toll-like receptors (TLRs), or intracellular signals, including alkylating DNA damage and oxidative stress [3]. AO/EB or annexin V/PI/7AAN dual staining could conveniently detect necrosis. Necrosis could be verified by inflammation, lactate dehydrogenase (LDH) release into the culture media, and cellular morphological changes, including cell swelling and cell membrane disruption [9].

GNP size affected the necrosis level. A systematic investigation of water-soluble GNPs (0.8–15 nm) determined that 1–2 nm-sized GNPs induced necrotic and apoptotic cell death, while the smaller or larger ones did not [75]. Cytotoxicity induced by GNPs in human bone marrow mesenchymal stem cells (hBMSCs) was size-dependent [76], with medium-sized GNPs (18.1 ± 2.1 nm) inducing cell death mostly through necrosis. In an in vivo study, hepatocyte injury (atrophy and necrosis) induced by GNPs (10, 20, and 50 nm) in rats was also size dependent, with the smallest GNPs inducing the highest effects [77]. Administration of 30 ppb of 18 nm-sized GNPs for 14 consecutive days in hamsters induced mild to marked degeneration and necrosis of hepatocytes and tubular epithelium [78], although no effects on plasma proteins, liver, and renal function were observed. Taken together, these data suggest that necrosis induced by GNPs is size dependent. Careful selection of size is needed for clinical application of GNPs, including the treatment of stem cell- and liver-related diseases.

**Figure 2:**
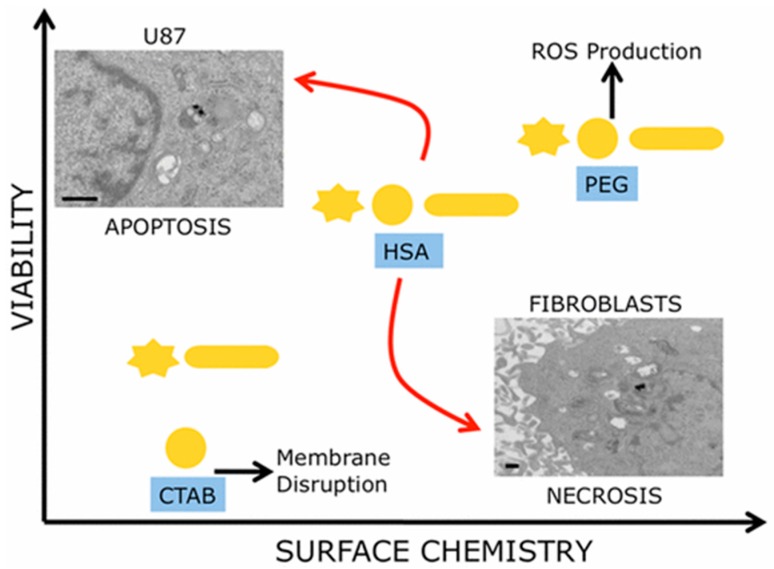
Surface chemistry of GNPs plays a predominant role in cell death dictation instead of size and shape. Reprinted with permission from reference [79]. Copyright (2017) American Chemical Society.

When GNP size, shape, and surface chemistry were considered to provide a multiparametric evaluation of their biocompatibility, it was observed that surface chemistry of the nanoparticles had predominant effects on cell proliferation (Figure 2) [79]. In this study, three different GNP morphologies (nanospheres, nanorods, and nanostars (GNSRs)), with each having three different surface decorations (CTAB, PEG, and human serum albumin (HSA)), were used to test cell viability of human glioblastoma and human dermal fibroblast cell lines. As a result, CTAB-coated particles were observed to be the most toxic to cells, and PEG-coated particles were determined to be the least toxic. Moreover, cancerous cells and healthy cells showed different death patterns stimulated by these surface-modified particles, with cancer cells dying via apoptosis and healthy cells via necrosis.

Necrosis induced by GNPs is cell type-dependent. Mild necrosis via DNA damage was induced by NIR-responsive GNRs (CTAB-stabilized) in A549 cells, while cell viability of normal human lymphocytes was not affected by the combined treatment of GNRs and NIR irradiation [80]. Furthermore, CTAB-capped GNR treatment disrupted lysosomes only in cancer cells, rather than normal cells, triggering both apoptosis and necrosis [81]. As mentioned above, there are still controversial reports on the cytotoxicity of CTAB-coated gold nanorods to cancer cells and normal cells, therefore, further investigation is still needed. Recently, biosynthesized GNPs are found to be biocompatible with normal human cells while showing potent cytotoxicity against cancer cells by inducing cell cycle arrest, apoptosis, and necrosis, indicating their potential as selective anticancer agents [82,83,84,85].

The relationship between GNPs’ properties and necrosis were summarized in Table 2. As to the necrosis mechanisms induced by GNPs, though necrosis could be elicited through extracellular and intracellular signals, GNP-induced necrosis was primarily ascribed to their ability to elicit the ROS production. For example, GNP-induced necrosis in hBMSCs is accompanied with ROS production [76]. ROS generation induced by GNPs leads to hepatocyte necrosis in rats [77]. In cancer cells with decreased expression of intracellular glutathione (GSH), GNPs induced necrosis by elevating intracellular ROS [86,87]. In addition, the discovery of the GNR-induced necrosis mediated by the lysosomal-cathepsin B pathway provides a further understanding in the mechanisms of the GNR specific cytotoxicity against cancer cells [81].

## 5. GNP-Induced Autophagy

Autophagy is a self-degrading process, where unnecessary or dysfunctional cellular components are degraded by lysosomes or vacuoles and recycled. Thus, autophagy is generally considered a survival mechanism, even though its deregulation has been related to non-apoptotic cell death [88]. There are three defined types of autophagy: macro-autophagy, micro-autophagy, and chaperone-mediated autophagy. Growing evidence reveals that autophagy plays an important role in the pathogenesis of a variety of human diseases, including neurodegeneration [89], cancer [90], infection and immunity [91], aging [92], myopathies [18], and liver and heart diseases [93]. Autophagy can act as a protector to prevent various diseases, but autophagy malfunction leads to pathology and can be deleterious. For example, autophagy exerts tumor suppressor effects by removing damaged organelles and reducing chromosome instability [94]. Conversely, the cytoprotective mechanism of autophagy allows cancer cells to survive in conditions of metabolic stress [95] and resist anticancer treatments [96]. Considering the important role of autophagy in human diseases, it could be a novel therapeutic target for disease treatment. Developing autophagy regulators provides a new strategy for disease therapy.

A growing body of literature has shown that nanomaterials could be a new class of autophagy regulators [97], and a variety of experimental approaches have been applied to determine nanomaterial-induced autophagy. TEM is the current gold standard method to characterize double-membrane vacuole structures, and the only tool able to reveal the morphology of autophagic structures [98]. Therefore, TEM has been widely applied to measure autophagy induced by nanomaterials, including GNPs, by observing autophagosomes in cells [99,100]. Conversely, Atg8/LC3 is the most widely monitored protein for verification of nanomaterial-induced autophagy. Atg8/LC3 levels were mostly detected by Western blotting. In addition, various green fluorescent protein (GFP)-LC3 stably transfected cell lines have been constructed for high-throughput autophagic effect screening of a nanomaterial library [101] and confirmation of nanomaterial-induced autophagy [102]. Additional autophagy-related protein markers applied in nanomaterial-induced autophagy include p62, PI3K, Akt, mTOR, Beclin 1, and Atg5 [97]. Nanomaterials may induce autophagosome accumulation either by blocking autophagic flux or inducing autophagy. Autophagosome/lysosome degradation inhibitors, such as bafilomycin A1 [101] and chloroquine [103], could be co-added with nanomaterials to distinguish between these possibilities.

**Figure 3:**
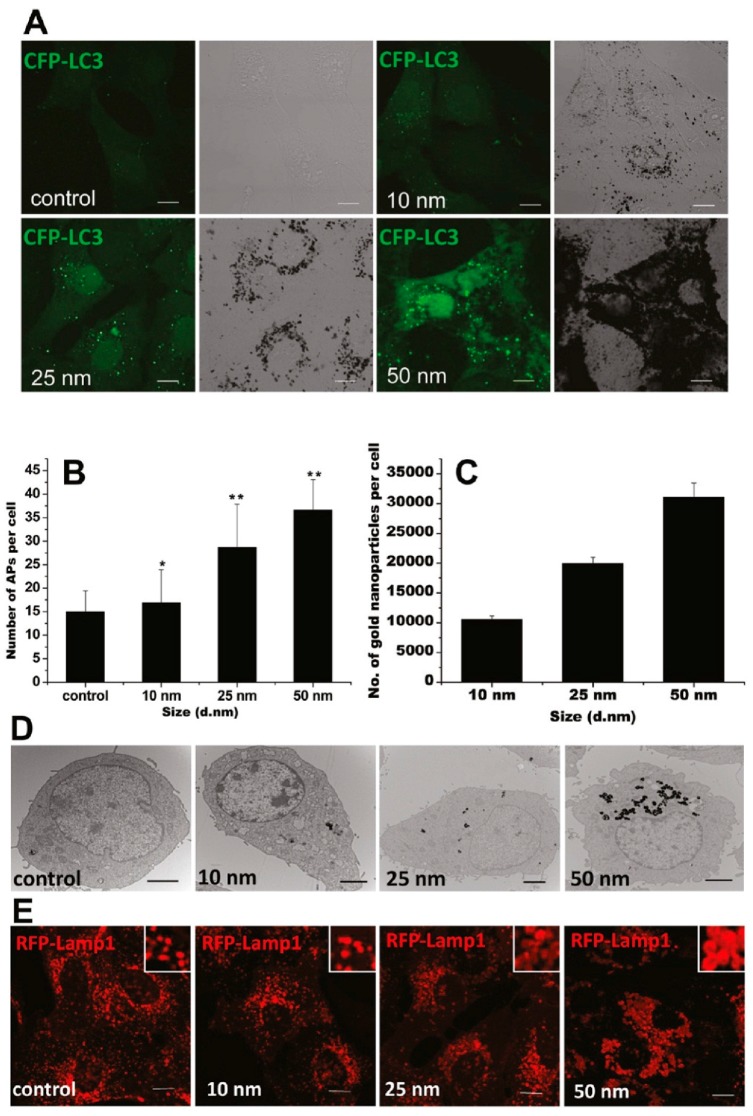
GNPs induce autophagosome accumulation through size-dependent nanoparticle uptake and lysosome impairment. (**A**) Formation of cyan fluorescent protein (CFP)-LC3 dots (pseudocolored as green) in CFP-LC3 NRK cells treated for 24 h with 1 nM GNPs of the sizes indicated. Left, confocal image; right, bright-field image (scale bar, 10 μm). (**B**) Statistical analysis of the number of autophagosomes (APs) per cell after 24 h of treatment. (**C**) Size-dependent cellular uptake of GNPs. The graph shows the number of GNPs per cell after incubation with 1 nM GNPs for 24 h. (**D**) TEM images of NRK cells untreated (control) or treated with GNPs with diameters of 10, 25, and 50 nm. The GNPs are internalized by cells and trapped inside lysosomes (scale bar, 2 μm). (**E**) Vacuoles induced by GNP treatment are enlarged lysosomes. NRK cells were incubated for 24 h with plain medium (control) or with 1 nM GNPs. Inset: close-up of the enlarged lysosomes (scale bar, 10 μm). Adapted with permission from reference [103]. Copyright (2011) American Chemical Society.

GNPs could induce autophagosome accumulation in cells [100], as modulated by their physicochemical properties, such as size, shape, surface chemistry, and dispersity. A recent study has shown that the GNP-induced formation of autophagosomes was size-dependent, in accordance with size-dependent GNP uptake and lysosome impairment (Figure 3) [103]. Moreover, compared to 13-nm-sized GNPs, 5 nm-sized GNPs were shown to be more toxic to hypoxic human renal proximal tubular cells (HK-2), as indicated by increased apoptosis and autophagic cell death [104]. On the contrary, 13 and 45 nm-sized GNPs were reported to induce autophagy in human periodontal ligament progenitor cells at 10 μM, but 5 nm-sized GNPs did not [105]. Considering the GNPs used in these two studies were synthesized via a similar NaBH_4_ reduction method, the surfaces of these GNPs were alike. The opposing results might be mainly attributed to the different cell lines.

Rod-shaped GNPs or gold nanorods can be taken up by the cells via endocytosis, translocated to the vesicular system, and expelled by cell exocytosis [106]. The few GNRs that escape from the vesicular system can be recycled back into lysosomes by cytoprotective autophagy. GNRs coated with CTAB induced significant autophagy in a dose-dependent manner in various cell lines of both tumor cells (HCT116, Huh7, PC3, Hela, and BEL7402) and non-malignant transformed cells (HEK293T, L02, and HFF). The CTAB-GNR-induced autophagy in HCT116 cells was Akt-independent [107]. While CTAB-GNRs with different aspect ratios (1, 2, 3, and 4) showed similar abilities to induce autophagy, surface modification of CTAB-GNRs with polystyrene sulfonate, poly allylamine hydrochloride, or both, dramatically decreased their autophagy induction abilities. These findings suggested that surface chemistry, but not aspect ratios, played a key role in determining the autophagy induction ability of GNRs. This result was further confirmed in another study, showing that CTAB-GNRs induced remarkable levels of autophagy activity in A549 cells, while polystyrene sulfonate- and poly(diallyldimethylammonium chloride)-coated GNRs barely induced autophagy. Therefore, GNRs with suitable decoration may be potentially used in lung cancer treatment [108]. Similarly, surface chemistry modulated the autophagic effect of gold nanospikes (GNSs). For example, surface modification with amines (NH_2_-GNSs), folic acid (FA) (FA-GNSs), and the cell-penetrating peptide TAT (TAT-GNSs) significantly increased cellular uptake of GNSs compared to unmodified methoxyl GNSs. This finding resulted in enhanced autophagy in human mouth epidermal carcinoma (KB) cells, as indicated by the upregulation of autophagy-related protein LC3-II and the accumulation of autophagosomes. Therefore, GNSs with delicate decoration could be used to treat mouth carcinoma [109]. 

Aggregated GNPs were demonstrated to be more effective at inducing autophagy than well-dispersed ones. For example, meso-2,3-dimercaptosuccinic acid-coated GNPs (12 nm) and sodium 2,3-dimercapto-1-propanesulfonate-coated GNPs (12 nm) dispersed in cell culture medium without pipetting would aggregate and precipitate on the surface of cells, inducing autophagy [102]. Meanwhile, pipetting could improve the dispersity of these GNPs, and therefore diminish such autophagic effects. Moreover, conjugation of GNPs with cancer therapeutic agents, such as snake venom protein toxin NKCT1 (purified *Naja kaouthia* protein toxin) [110], chloroquine [111], and tumor necrosis factor-related apoptosis-inducing ligand [112], enhanced anticancer activity of these drugs in various kinds of cancer cells by inducing autophagic cell death, providing potential chemotherapeutic strategies for cancer treatment.

GNP-induced autophagy in mammalian cells can also be cell type-dependent. In one study, GNP-induced cell growth inhibition was studied in human lung fibroblasts (MRC-5), mouse fibroblasts (NIH3T3), porcine kidney epithelial cells (PK-15), and African green monkey kidney epithelial cells (Vero) [72]. Results showed that commercially available GNPs induced autophagic attenuation of cell growth only in NIH3T3 cells. In another study, HK-2 cells under hypoxic conditions were reported to be more susceptible to GNP (5 nm) exposure compared to that of normoxic cells [104]. While exposure to 5 nm-sized GNPs caused autophagy and cell survival in normoxic HK-2 cells, GNP exposure under the same conditions increased ROS production, led to the loss of mitochondrial membrane potential, and resulted in increased apoptosis and autophagic cell death in hypoxic cells. These results also agreed with the observation that cellular uptake of GNPs in hypoxic cells was considerably higher than that in normoxic cells. In addition, cell microenvironments can alter the physical properties of GNP–drug conjugates and influence their capabilities in inducing cellular autophagy. For example, GNPs conjugated with Rad6 inhibitor SMI#9 (SMI#9-GNP) was shown to be cytotoxic in mesenchymal triple negative breast cancer (TNBC) subtype (SUM1315 and MDA-MB-231) cells, but not in basal TNBC subtype (MDA-MB-468 and HCC1937) cells or normal breast cells, as indicated by induction of apoptosis, autophagy, and necrosis [113]. Aggregation of SMI#9-GNP at the surface of basal TNBC subtype cells, but not mesenchymal TNBC subtype cells, contributed to the decreased toxicity seen in basal TNBC subtype cells.

As a new type of autophagy modulator, GNPs may affect autophagy through various mechanisms. Oxidative stress has been considered one of the major mechanisms of GNP-induced cytotoxicity and has been hypothesized to play a remarkable role in the modulation of autophagy. Treatment of cells with GNPs [100], GNRs [107], and GNSs [109] resulted in high ROS generation, which can have a complex interaction with autophagy. Indirectly, activation of the AMPK pathway due to elevated levels of ROS led to inhibition of the mTOR pathway, resulting in activation of autophagy [114]. On the other hand, the rise in ROS directly oxidized and inactivated Atg4, leading to Atg8 lipidation and autophagy induction [115]. In addition, mitochondrial damage from ROS production contributed to the induction of autophagy [107,110]. As most GNPs enter the cell through endocytosis, accumulation of GNPs in lysosomes may directly cause their impairment and result in autophagosome accumulation. For example, treatment with GNPs caused lysosome alkalinization, leading to impairment of autophagosome/lysosome fusion and reduced lysosome degradation capacity, ultimately resulting in autophagy blockage [103]. 

In summary, GNPs can cause autophagosome accumulation in various types of cell lines via either inducing autophagy or blocking autophagic flux. On one hand, autophagy can be a survival mechanism for cells in response to cellular damage caused by GNPs. On the other hand, GNPs may impair lysosome functions, resulting in autophagic cell death. Autophagic effects can be modulated by regulating the physicochemical properties of GNPs (Table 3). In addition, GNP-induced autophagy varies by cell type. The possible mechanisms of GNP-induced autophagy include oxidative stress, mitochondrial damage, and impairment of the autophagosome/lysosome system due to accumulation of GNPs in the lysosomes. Further studies should investigate medical applications of GNPs for the treatment of autophagy-related diseases.

## 6. Conclusions and Perspectives for Future Work

Cell death, especially apoptosis, necrosis, and autophagy, is crucial to human health. Unregulated cell death is associated with fatal diseases. Therefore, powerful regulators of unregulated cell death are urgently needed for disease treatment. Due to the stability and decoration convenience, GNPs are frequently used in nanomedicine and disease treatment, especially in drug delivery and photothermal therapy under external stimulation. Based on reported literature, GNPs were also observed to induce diverse types of cell death, primarily through intrinsic pathways, including ROS production, mitochondrial function disturbance, and ER stress. GNP size, shape, surface chemistry, and dispersion state play important roles in cell death dictation. Small sized GNPs are more liable to induce necrosis. Compared to spherical-shaped GNPs, hexagonal GNPs and GNRs are more liable to induce apoptosis. Hydrophobic and charged GNPs induced higher apoptosis and autophagy levels than hydrophilic and neutral charged GNPs, respectively. Aggregated GNPs are more effective at inducing autophagy than well-dispersed ones. Therefore, GNPs may act as new cell death regulators and show potential application in the treatment of various diseases through dictation of GNP properties, including cancer treatment. As we all know, the contents released by necrotic cells are highly inflammatory, which will invariably promote inflammation in the body, leading to the promotion and migration of tumors [116]. Since apoptosis evokes minimal or no inflammation of surrounding tissues, intervention by GNPs to switch cell fate towards apoptotic death could be beneficial in cancer treatment. Moreover, targeting ligands decorated on GNPs make the cell death dictation of targeting cells and tissues more efficient [117,118].

Except for experimental methods, quantitative nanostructure-activity relationship (QNAR) modeling could also be employed to predict cell death induced by GNPs. For the diverse cell death modalities, these types of cell death may occur simultaneously and interplay with each other. However, most papers have only focused on one type of cell death. Therefore, the determination of diverse cell death types by GNP properties should also be investigated in the future.

## Figures and Tables

**Table 1:** The relationship between gold nanoparticles (GNPs)’ properties and apoptosis.

	Properties		Cell Type		Cell Death			Ref.
	Size	Surface	Normal	Cancer	Apoptosis	Necrosis	Autophagy	
**GNPs**	1 nm	BSA		HepG-2	+	+	N.A.	[65]
	1.5 nm	TMAT (positive charge), MES (negative charge)	HaCaT		+	−	N.A.	[55]
	2.3 nm	l/d-glutathione		MGC-803	+(d-glutathione > l-glutathione)	−	N.A.	[58]
	2.7 nm	2-mercapto-1-methylimidazole		SH-SY5Y	+	N.A.	N.A.	[66]
	1–10 nm	sodium polyacrylate (negative charge)	freshly isolated human neutrophils		+	N.A.	N.A.	[56]
	7.4 nm	PVP		HT-29	+	−	N.A.	[59]
	13 nm	citrate	Primary rabbit articular chondrocytes		+	−	N.A.	[52]
	13 nm	PEG	Liver of BALB/c mice		+	N.A.	N.A.	[67]
	20–25 nm	DCaLC, DCaC, DcaDC Hydrophobicity: DCaLC > DCaDC > DCaC		A549	+(DCaLC > DCaDC > DCaC)	N.A.	N.A.	[57]
	29 nm	NaBH_4_		A549	+	N.A.	N.A.	[64]
	40 nm	citrate	head kidney of *Sparus aurata*		+	N.A.	N.A.	[63]
	40 nm	PVP	head kidney of *Sparus aurata*		+	N.A.	N.A.	[63]
	10–40 nm	N.A.	Vero cells		+	N.A.	N.A.	[72]
	4 nm, 100 nm	PEG	liver tissue of BALB/c mice		+(4 nm ≈ 100 nm)	N.A.	N.A.	[51]
	8, 15, 40 nm	citrate	SPEV, HL7702	HT-29, A549	+	+	N.A.	[60,61,62]
	5 nm, 15 nm	citrate	human peripheral blood lymphocytes (PBL), Raw264.7		+(15 nm > 5 nm)	+	N.A.	[53]
	20 nm, 70 nm	N.A.	Human polymorpho-nuclear neutrophil cells		+	N.A.	N.A.	[74]
**GNRs**	16.7 nm × 43.8 nm	PEG	HaCaT		+	N.A.	N.A.	[50]
	10 nm × 39 nm, 10 nm × 41 nm	N.A.		AGS	+	N.A.	N.A.	[71]
	50–60 nm × 20–30 nm	CTAB		MCF-7, N87	+	+	N.A.	[73]
**Hexagonal GNPs**	150 nm × 9 nm	HS–C_2_H_4_–CONH–PEG–O–C_3_H_6_–COOH		Calu-3	+	N.A.	N.A.	[54]

+: positive results; −: negative results; N.A.: not available.

**Table 2:** The relationship between GNPs’ properties and necrosis.

	Properties		Cell Type		Cell Death			Ref.
	Size	Surface	Normal	Cancer	Apoptosis	Necrosis	Autophagy	
GNPs	1–2 nm	triphenylphosphine monosulfonate (TPPMS); tris-sulfonated triphenylphosphine (TPPTS)	L929; J774 A1	HeLa; SK-Mel-28	+	+	N.A.	[75]
	10 nm	N.A.	Rat hepatocyte		N.A.	+	N.A.	[77]
	14.6 nm	polyphenols		MCF-7	N.A.	+	N.A.	[85]
	18 nm	citrate	Hamsters hepatocyte	N.A.	N.A.	+	N.A.	[78]
	18.1 nm	citrate	2M-125C	N.A.	+	+	N.A.	[76]
	20 nm	morin	PBMCs, HBL-100 cells		−	−	N.A.	[82]
	20 nm	morin		MCF-7	+	+	N.A.	[82]
	25.1 nm	CTAB	human dermal fibroblast (hfb) cells		−	+	N.A.	[79]
	25.1 nm	CTAB		U87	+	−	N.A.	[79]
	25.1 nm	HSA	human dermal fibroblast (hfb) cells	U87	−	−	N.A.	[79]
	37 nm	N.A.		A549	+	−	N.A.	[83]
	50 nm	polyphenols		A431	+	+	N.A.	[84]
GNRs	52.7 nm × 23.0 nm	CTAB	human dermal fibroblast (hfb) cells		−	+	N.A.	[79]
	52.7 nm × 23.0 nm	HSA	human dermal fibroblast (hfb) cells	U87	−	+	N.A.	[79]
	119 × 28 nm	CTAB	normal human blood lymphocytes		−	−	N.A.	[80]
	119 × 28 nm	CTAB		A549	−	+	N.A.	[80]
	20 × 53 nm	CTAB	CHO		−	−	N.A.	[81]
	20 × 53 nm	CTAB		MCF-7	+	+	N.A.	[81]
GNSRs	The tip-to-tip distance: 62.5 ± 11.2 nm	CTAB	human dermal fibroblast (hfb) cells		−	+	N.A.	[79]
	The tip-to-tip distance: 62.5 ± 11.2 nm	CTAB		U87	+	−	N.A.	[79]
	The tip-to-tip distance: 62.5 ± 11.2 nm nm	HSA	human dermal fibroblast (hfb) cells		−	+	N.A.	[79]
	The tip-to-tip distance: 62.5 ± 11.2 nm nm	HSA		U87	−	−	N.A.	[79]

+: positive results; −: negative results; N.A.: not available.

**Table 3:** The relationship between GNPs’ properties and autophagy.

	Properties		Cell Type		Cell Death			Ref.
	Size	Surface	Normal	Cancer	Apoptosis	Necrosis	Autophagy	
GNPs	5 nm	citrate	PDLPs		N.A.	N.A.	bloakade of autophagy	[105]
	5 nm	citrate	HK-2		+ (only in hypoxic cells)	N.A.	+ (in hypoxic cells > in normoxic cells)	[104]
	20 nm	citrate	MRC-5		N.A.	N.A.	+	[100]
	10, 25, 50 nm	citrate	NRK		N.A.	N.A.	+ (blockade of autophagic flux, 50 nm > 25 nm > 10 nm)	[103]
	13, 45 nm	citrate	PDLPs		N.A.	N.A.	+ (45 nm > 13 nm)	[105]
GNRs	N.A.	CTAB		HCT116, BEL7402	+	N.A.	+	[107]
	N.A.	CTAB	HEK293T, L02, HFF	PC3	N.A.	N.A.	+	[107]
	N.A.	CTAB/PSS, CTAB/PAH, CTAB/PSS/PAH, CTAB/PAH/PSS		HCT116	−	N.A.	−	[107]
	55 nm × 14 nm (length × diameter)	CTAB		A549	N.A.	N.A.	+	[108]
		PSS, PDDAC		A549	N.A.	N.A.	−	[108]
	average length of 10–40 nm	N.A.	Vero		+	N.A.	N.A.	[72]
	average length of 10–40 nm	N.A.	NIH3T3		−	N.A.	+	[72]
	average length of 10–40 nm	N.A.	MRC-5		−	N.A.	N.A.	[72]
GNSs	54 ± 9 nm	no coating (GNSs), HS-PEG-NH_2_ (NH_2_-GNSs), HS-PEG-FA (FA-GNSs), cysteine-terminated TAT (TAT-GNSs)		KB	+ (TAT-GNSs > FA-GNSs > NH2-GNSs > GNSs)	N.A.	+ (TAT-GNSs > FA-GNSs > NH2-GNSs > GNSs)	[109]

+: positive results; −: negative results; N.A.: not available.
